# Disease-Specific Survival in *De Novo* Metastatic Renal Cell Carcinoma in the Cytokine and Targeted Therapy Era

**DOI:** 10.1371/journal.pone.0063341

**Published:** 2013-05-03

**Authors:** Sumanta K. Pal, Rebecca A. Nelson, Nicholas Vogelzang

**Affiliations:** 1 Department of Medical Oncology & Experimental Therapeutics, City of Hope Comprehensive Cancer Center, Duarte, California, United States of America; 2 Division of Biostatistics, Department of Information Science, City of Hope Comprehensive Cancer Center, Duarte, California, United States of America; 3 US Oncology Research, Comprehensive Cancer Centers, Las Vegas, Nevada, United States of America; University Clinic of Navarra, Spain

## Abstract

**Background:**

Recent phase III studies of targeted agents for metastatic renal cell carcinoma (mRCC) have generated median survival estimates that far exceed those observed during the cytokine era. However, substantial population-based data does not exist to confirm this trend. We sought to determine whether survival has improved for patients with mRCC diagnosed in the era of targeted therapies, as compared to the era of immunotherapy.

**Methods:**

The Surveillance, Epidemiology, and End Results (SEER) Registry was used to identify patients aged 18 and older diagnosed stage IV RCC between 1992 and 2009. Patients had documented clear cell, papillary or chromophobe histology. The Kaplan Meier method and log-rank test were used to compare disease-specific survival (DSS) for patients diagnosed from 1992–2004 (i.e., the cytokine era) and 2005–2009 (i.e., the targeted therapy era). Univariate and multivariate analyses of relevant clinicopathologic characteristics were also performed.

**Results:**

Of 5,176 patients identified using the above characteristics, 2,392 patients were diagnosed from 1992–2004 and 2,784 from 2005–2009. Median DSS was improved in those patients diagnosed from 2005–2009 (16 months *vs* 13 months; P<0.0001). A similar temporal trend towards improving survival was noted in patients with clear cell (P = 0.0006), but not in patients with non-clear cell disease (P = 0.32). Notable findings on multivariate analysis include an association between shorter DSS and the following characteristics: (1) diagnosis from 1992–2004, (2) advanced age (80+), and (3) absence of cytoreductive nephrectomy.

**Conclusions:**

These data reflect progress in the management of mRCC, specifically in the era of targeted therapies. Notably, it was inferred that certain treatment strategies were employed during pre-specified time periods, representing a major caveat of the current analysis. Further studies related to the influence of age and race/ethnicity are warranted, as are studies exploring the role of cytoreductive nephrectomy and novel treatments for non-clear cell disease.

## Introduction

The treatment paradigm for metastatic renal cell carcinoma (mRCC) has undergone a dramatic evolution over the past two decades. In 1992, interleukin-2 (IL-2) was approved for the treatment of mRCC. Although IL-2 has been shown to lead to durable responses in a small proportion of patients, the vast majority of patients either derive no clinical benefit or are physically too debilitated to receive this intensive therapy [1]. As an alternative, monotherapy with interferon-α (IFN-α) was frequently employed. A meta-analysis of data from IFN-α trials showed modest results at best, with a median time to progression (TTP) of 4.7 months and a median overall survival (OS) of 13 months [2]. At the time these data were published in 2002, it was suggested that IFN-α serve as a reference standard for future clinical trials in mRCC.

The introduction of targeted therapies for mRCC shattered this reference standard. A total of seven targeted agents have been approved to date by the US FDA on the basis of phase III data – four vascular endothelial growth factor-tyrosine kinase inhibitors (VEGF-TKIs; sunitinib, sorafenib, pazopanib, and axitinib), one VEGF-directed monoclonal antibody (bevacizumab), and two inhibitors of the mammalian target of rapamycin (mTOR; temsirolimus and everolimus) [3], [4], [5], [6], [7], [8], [9]. With the advent of these therapies, IL-2 and IFN-α are presumably utilized to a lesser extent in the mRCC paradigm.

It has been repeatedly observed that survival in more recent trials in mRCC has gone far beyond the landmark of 13 months proposed in association with IFN-α. For instance, in the randomized phase III study comparing sunitinib and IFN-α in treatment-naïve patients, a median OS of 26.4 months was observed with sunitinib therapy [4]. Long-term survivors are also increasingly recognized with targeted therapy; in a phase II study of axitinib, up to 20% of patients were still alive 5 years beyond the time of treatment initiation [10].

Although these data provide compelling rationale to suggest that survival has improved since the advent of targeted therapies, this hypothesis has not been definitively proven. In the current study, we queried the Survival, Epidemiology, and End Results (SEER) dataset and performed generational analysis of survival amongst patients with mRCC. With data extending from 1983 to 2009, we segregated our analysis using two clinically relevant time points: (1) the approval of IL-2 in 1992 and (2) the approval of the first targeted therapies (sunitinib and sorafenib) in 2004 [11], [12].

## Methods

### Patient Selection

The SEER dataset was analyzed for the current study, a registry encompassing approximately 28% of the US population [13]. The SEER Program has extensive data pertaining to demographics, stage, tumor histology, and grade. The current analysis was restricted to patients 18 and older who had a diagnosis of RCC between 1992 and 2009 (n = 60,587). The analysis was further limited to patients with stage IV disease at the time of diagnosis, had a known surgical status, had a known cause of death if deceased, and had a clinically relevant histology (n = 5,150). Notably, the SEER Registry does not allow for capture of patients who progressed from localized or regional disease to metastatic disease, thus confining this analysis to those with *de novo* metastatic disease.

### Tumor Classification

Reporting of tumor histology, grade and stage was done in accordance with the International Classification of Diseases for Oncology (ICD-O), version 3. Given the heterogeneity of histologic classification of kidney tumors in SEER, we limited our analyses to the three most clinically relevant histologic subtypes – clear cell (ICD-O 8310), papillary (ICD-O 8260), and chromophobe (ICD-O 8317). In our analyses, papillary and chromophobe tumors were combined in a category termed ‘non-clear cell’. Tumor grade was characterized as well differentiated, moderately differentiated, poorly differentiated or undifferentiated (a Fuhrman grade is not delineated in the SEER Registry). Stage IV disease was identified according to criteria specified within the American Joint Committee on Cancer (AJCC) TNM staging system, 7^th^ ed [14].

### Statistical Analysis

To test the *a priori* hypothesis that patients diagnosed in the targeted therapy era have a longer survival as compared to patients diagnosed in the cytokine era, we assessed our primary outcome, disease-specific survival (DSS), across two time periods: (1) 1992–2004, and (2) 2005–2009. These time periods reflect (1) the approval of IL-2 in 1992, and (2) the first approval of a targeted therapy for mRCC (sorafenib) in 2005 [12]. Clinicopathologic characteristics were compared between the groups using the student's t-test and chi-square test for continuous and categorical variables, respectively. Disease-specific survival was assessed, and defined as the time elapsed between date of diagnosis with RCC and date of death, if attributable to RCC. Patient data were censored at the time of last follow-up if the patient was still alive at last contact. Patients with an unknown cause of death were excluded. The Kaplan-Meier method and log-rank test were used to compare survival across the two time periods. Relevant clinicopathologic characteristics were evaluated for their association with disease specific survival using both univariate and multivariate Cox proportional hazard models.

After a thorough review of the SEER methodology, we felt it appropriate to exclude T-stage and N-stage from our analyses. Specifically, in 2004 SEER adopted the Collaborative Stage Data Collections System (CS). Prior to CS, patients with M1 stage disease were rarely coded with specifics on T- and N-stage other than Tx and Nx, respectively [15]. Hierarchical rules in SEER classification implied that T- and N-stage were “trumped” by M1 status. In contrast, from 2004 onwards, rules were set in place that called for recording of T-stage and N-stage despite the notation of M1 disease [16].

All analyses were performed using SAS (SAS Institute, Cary, NC, USA). P-values reported herein are two-sided. P-values of 0.05 or less were deemed statistically significant.

## Results

### Patient characteristics by time period

Utilizing the aforementioned selection criteria, a total of 2,382 patients were identified from 1992–2004 and 2,768 patients from 2005–2009. Characteristics of the study population are noted in Table 1. The mean age was similar across the two study periods (approximately 62 for both) and, as anticipated, a male preponderance was observed in both groups. A greater representation of minority groups was seen in the latter study period as compared to the earlier study period, with an increased proportion of blacks (8.0% *vs* 7.5%) and Hispanic whites (12.4% *vs* 15.0%). A larger proportion of patients in the latter study period were noted to have poorly differentiated or undifferentiated tumors (69.0% *vs* 58.7%). Nephrectomy rates were similar across time periods, with 60.0% of the study population receiving this intervention. As expected, the duration of follow-up was substantially shorter for those patients assessed in the later time period as compared to the earlier time period (14 months and 24.5 months, respectively; P<0.0001).

**Table 1:** Patient characteristics by treatment period.

		1992–2004 N (%)	2005–2009 N (%)	p-value
Age	Mean (±SD)	62.3 (±11.8)	62.2 (±11.7)	0.7993
Age Group	18–49	339 (14.2%)	371 (13.4%)	0.4818
	50–64	1032 (43.3%)	1249 (45.1%)	
	65–79	839 (35.2%)	938 (33.9%)	
	80+	172 (7.2%)	210 (7.6%)	
Sex	Men	1592 (66.8%)	1887 (68.2%)	0.3067
	Women	790 (33.2%)	881 (31.8%)	
Race/Ethnicity	Non-Hispanic White	1747 (73.3%)	1943 (70.2%)	0.0188
	Black	179 (7.5%)	221 (8%)	
	Hispanic White	295 (12.4%)	414 (15%)	
	API§	125 (5.2%)	155 (5.6%)	
	AI/AN¥	28 (1.2%)	19 (0.7%)	
	Unknown	8 (0.3%)	16 (0.6%)	
Histology	Clear Cell	2194 (92.1%)	2491 (90%)	0.0083
	Non-Clear Cell	188 (7.9%)	277 (10%)	
Grade	Well Differentiated	81 (3.4%)	94 (3.4%)	<.0001
	Moderately Differentiated	421 (17.7%)	490 (17.7%)	
	Poorly Differentiated	532 (22.3%)	838 (30.3%)	
	Undifferentiated	183 (7.7%)	461 (16.7%)	
	Unknown	1165 (48.9%)	885 (32%)	
Surgery Type	No Nephrectomy	981 (41.2%)	1077 (38.9%)	0.0966
	Nephrectomy	1401 (58.8%)	1691 (61.1%)	

§ Asian-Pacific Islander.

¥ American Indian/Alaskan Native.

### Analysis of survival by time period

**Figure 1:**
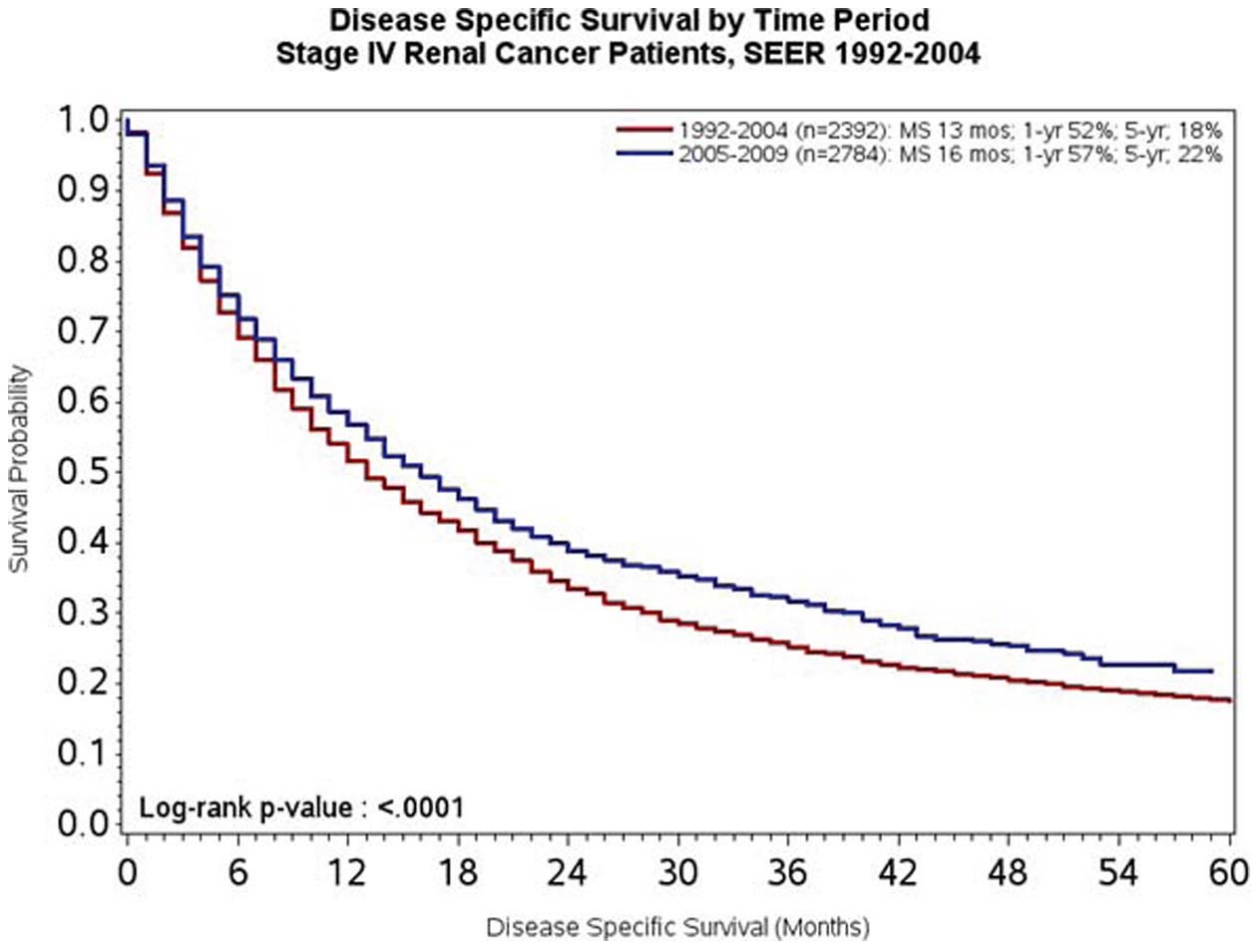
DSS of patients with *de novo* mRCC diagnosed from 1992–2004 as compared to 2005–2009.

**Figure 2:**
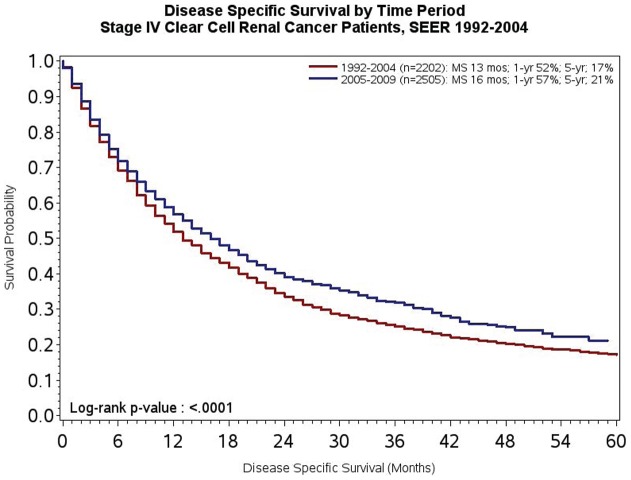
DSS of patients with *de novo* mRCC diagnosed from 1992–2004 as compared to 2005–2009 with clear cell disease.

**Figure 3:**
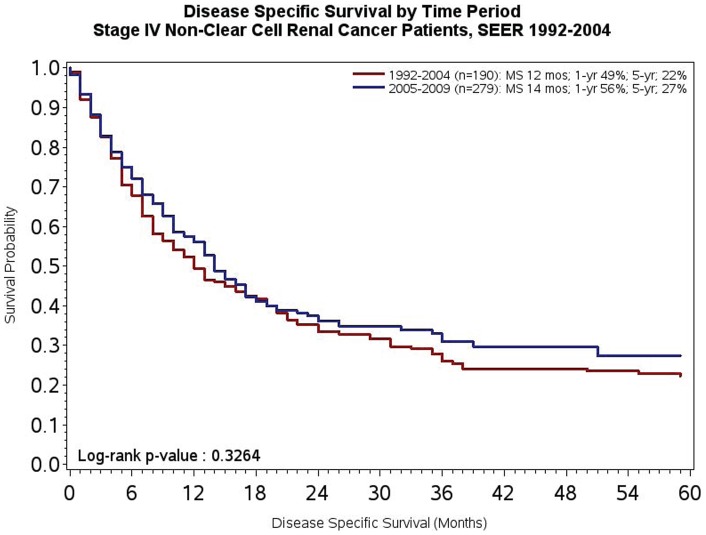
DSS of patients with *de novo* mRCC diagnosed from 1992–2004 as compared to 2005–2009 with non-clear cell (i.e., papillary or chromophobe) disease.

To test the *a priori* hypothesis of this work, DSS was compared across the two time periods of interest. As noted in Figure 1, median DSS was 13 months in patients diagnosed from 1992–2004 compared to 16 months in patients diagnosed between 2005–2009 (P<0.0001). At both 1-year and 5-year landmarks, survival also appeared to be superior amongst patients diagnosed between 2005–2009 as compared to patients diagnosed between 1992–2004 (57% *vs* 52% at 1-year, and 22% *vs* 18% at 5-years, respectively). Given that the majority of systemic therapies have been assessed in clear cell mRCC, we then compared survival in clear cell and non-clear cell subsets. As noted in Figure 2, reflecting the substantial proportion of patients with clear cell disease, the survival trends were akin to those observed in the overall study population. Amongst patients with non-clear cell disease, as was anticipated, no significant difference in DSS was observed (P = 0.32; Figure 3).

### Univariate and multivariate analysis

As shown in Table 2, univariate analyses demonstrated a number of factors beyond date of diagnosis that were associated with DSS. Relative to patients diagnosed aged 18–49, older patients (specifically, patients between 65–79 and ≥80) had shorter survival. Amongst clinicopathologic criteria, poorly differentiated or undifferentiated tumors were associated with shorter survival; no significant difference was noted in survival based on histology (i.e., clear cell *vs* non-clear cell). Survival was improved in patients who had received cytoreductive nephrectomy as compared to those who had not. On multivariate analysis, time period was independently associated with survival, favoring patients diagnosed from 2005–2009 (HR 0.88, 95% CI 0.83–0.94; P = 0.0001). Complete results of multivariate analysis are displayed in Table 2; as noted therein, older age, female sex and black race were amongst the clinical characteristics independently associated with shorter survival. Higher tumor grade and absence of nephrectomy were also independently associated with shorter survival.

**Table 2:** Univariate and multivariate analysis of DSS.

			Univariate		Multivariate	
		N (%)	Hazard Ratio (95% CI)	p-value	Hazard Ratio (95% CI)	p-value
Time Period	1992–2004	2382 (46.3%)	1.00 (reference)	–	1.00 (reference)	–
	2005–2009	2768 (53.7%)	0.87 (0.81–0.93)	<.0001	0.87 (0.81–0.93)	<.0001
Age	Mean (±SD)	62.2 (±11.7)	1.01 (1.01–1.02)	<.0001	–	–
Age Group	18–49	710 (13.8%)	1.00 (reference)*	–	1.00 (reference)	–
	50–64	2281 (44.3%)	1.05 (0.95–1.17)	0.3177	1.04 (0.94–1.16)	0.4114
	65–79	1777 (34.5%)	1.21 (1.09–1.35)	0.0004	1.05 (0.95–1.18)	0.3400
	80+	382 (7.4%)	1.62 (1.39–1.88)	<.0001	1.16 (1.00–1.36)	0.0541
Sex	Men	3479 (67.6%)	1.00 (reference)	–	1.00 (reference)	
	Women	1671 (32.4%)	1.23 (1.14–1.32)	<.0001	1.21 (1.13–1.30)	<.0001
Race/Ethnicity	Non-Hispanic White	3690 (71.7%)	1.00 (reference)	–	1.00 (reference)	–
	Black	400 (7.8%)	1.11 (0.98–1.26)	0.1023	1.02 (0.90–1.16)	0.7880
	Hispanic White	709 (13.8%)	0.99 (0.89–1.09)	0.7777	0.95 (0.86–1.06)	0.3670
	API§	280 (5.4%)	0.75 (0.64–0.88)	0.0004	0.78 (0.67–0.92)	0.0024
	AI/AN¥	47 (0.9%)	1.24 (0.91–1.71)	0.1779	1.07 (0.78–1.48)	0.6565
	Unknown	24 (0.5%)	0.81 (0.47–1.40)	0.4521	1.03 (0.60–1.78)	0.9168
Histology	Clear Cell	4685 (91%)	1.00 (reference)	–	1.00 (reference)	–
	Non-Clear Cell	465 (9%)	0.97 (0.86–1.09)	0.5624	1.02 (0.90–1.15)	0.7766
Grade	Well Differentiated	175 (3.4%)	1.00 (reference)	–	1.00 (reference)	–
	Moderately Differentiated	911 (17.7%)	0.93 (0.75–1.15)	0.4925	1.19 (0.96–1.47)	0.1054
	Poorly Differentiated	1370 (26.6%)	1.09 (0.89–1.34)	0.3929	1.59 (1.29–1.95)	<.0001
	Undifferentiated	644 (12.5%)	1.39 (1.12–1.72)	0.0025	2.13 (1.71–2.65)	<.0001
	Unknown	2050 (39.8%)	1.81 (1.49–2.21)	<.0001	1.38 (1.13–1.69)	0.0013
Surgery Type	No Nephrectomy	2058 (40%)	1.00 (reference)	–	1.00 (reference)	–
	Nephrectomy	3092 (60%)	0.37 (0.35–0.40)	<.0001	0.35 (0.32–0.38)	<.0001

* Trend p-value across age groups, p<0.0001.

§ Asian-Pacific Islander.

¥ American Indian/Alaskan Native.

## Discussion

The results described herein suggest that survival has improved in the era of targeted therapies as compared to the era of cytokine therapy. This suspicion has been strongly held in the academic community for some time, given the substantial improvement in overall survival seen in recent studies assessing systemic therapy in treatment-naïve populations. For instance, in the recently reported COMPARZ study assessing sunitinib and pazopanib in the front-line setting, a median OS of 28.4 months and 29.3 months was observed in each treatment arm, respectively [17]. These data stand in sharp contrast to estimates of survival generated a decade ago where, in the era of cytokine therapies, a median survival approaching 1 year was anticipated [18]. Our review of the SEER registry encompasses a highly heterogeneous array of patients, and suggests a similar survival trend in survival – diagnosis with stage IV RCC between 2005–2009, during which time several targeted therapies were approved by the US FDA (including sorafenib, sunitinib, and everolimus), is independently associated with improved survival. Another recent epidemiologic study utilizing data from the California Cancer Registry has pointed towards a similar improvement in survival; however, this study included non-metastatic patients and further capped analysis at 2007 [19]. The latter would likely limit the effect seen from targeted therapies, introduced from 2005 onwards.

The magnitude of difference in survival – from 13 months between 1992–2004 to 16 months between 2005–2009 – may not be interpreted as substantial progress. However, several caveats of our analysis must be accounted for. First, patients with metachronous metastatic disease are not captured in the SEER registry. Thus, our analysis is restricted to those patients with *de novo* metastatic disease. Across the commonly used classification schema for mRCC (i.e., MSKCC and Heng criteria, etc.), a time from diagnosis to initiation of systemic therapy less than 1 year is identified as adverse prognostic factor [18], [20]. Patients with *de novo* metastatic disease inherently possess this characteristic, and by the MSKCC or Heng risk stratification tools, this precludes them from having “good-risk” disease. Thus, our dataset is reflective of a population of intermediate- and poor-risk patients. The most recent risk stratification tool, developed by the International Kidney Cancer Working Group, includes a compilation of patient level data for 3,748 patients with mRCC enrolled in clinical trials between 1975 and 2002 [21], [22]. This dataset also identifies an abbreviated time from diagnosis to treatment as an adverse prognostic factor. Notably, in this most recent series, patients who started treatment within a window of 506 days of diagnosis (encompassing those patients with *de novo* metastatic disease) had a median survival of 1.31 years. These data, pooled over clinical trials conducted over a wide span of time, are similar to what we have observed in the current report. A recent update of this dataset, like ours, suggests a temporal trend towards improved survival [22]. However, it is important to keep in mind that their analyses included patients involved in clinical trials until 2002 – well before the widespread utilization of targeted therapies.

Several other key distinctions in the SEER data may explain the small magnitude of difference in survival observed in our study. When reflecting upon the aforementioned phase III studies in mRCC evaluating targeted agents, it is critical to keep in mind that stringent eligibility criteria were set in place for each of these studies. Patients included within the SEER registry, in contrast to those on clinical trials, were not limited by performance status, organ function, and comorbidity. A report from the International mRCC Database consortium suggested that approximately 43% of patients in their experience would not be candidates for clinical trials based on standard eligibility criteria (i.e. presence of brain metastases, performance status, etc.) [23]. Furthermore, patients in the SEER registry did not necessarily receive systemic therapy. Patients with poor global health and those that did not receive systemic treatments may account for the rather modest median DSS of 14 months noted from 2005–2009.

Outside of the finding that survival was improved in the era of targeted therapies, there were several other notable clinical and treatment-related characteristics associated with survival. Perhaps most notably, nephrectomy was independently associated with improved survival. Nephrectomies identified within this cohort inherently represent cytoreductive procedures, as all patients had *de novo* metastatic disease. Although its role is well established in the setting of cytokine therapy, cytoreductive nephrectomy is controversial in the era of targeted therapies [24]. A selection bias may confound the observed association with survival in the SEER dataset – patients with a poor performance status, greater comorbidity, or aggressive and invasive primary tumors are less likely to undergo surgery. Two ongoing studies, the French-led CARMENA study and a separate trial by the European Organization for the Research and Treatment of Cancer (EORTC), assess the role of cytoreductive nephrectomy as an adjunct to therapy with sunitinib for patients with mRCC [25], [26].

Other notable findings from our analysis include a shorter survival amongst black patients relative to non-Hispanic whites. These data point towards potential disparities in access to care, or perhaps to biological differences across ethnicity. Substantial research in this domain is currently lacking, although other reports allude to similar findings [27], [28]. A caveat of assessing the impact of race and ethnicity within the SEER database is that the catchment area of the database has evolved over time. Specifically, beginning in 2000, 6 new registries were added – great California, greater Georgia, Kentucky, Louisiana and New Jersey. If these areas had greater numbers of minorities, these could skew the results observed herein. With respect to pathologic characteristics, the finding of shorter survival amongst patients with poorly differentiated or undifferentiated tumors (as compared to well differentiated tumors) was expected. Also as anticipated, there was no substantial improvement in survival amongst patients with non-clear cell (specifically, papillary or chromophobe) histology. The vast majority of phase III studies assessing targeted agents (with the notable exception of the pivotal study assessing temsirolimus) required the presence of clear cell disease [5]. To date, no assessment has been made as to whether or not the therapeutic advances made amongst patients with clear cell mRCC are applicable to those with papillary or chromophobe disease. Prospective efforts to characterize the activity of sunitinib in papillary mRCC, for instance, have yielded disappointing response rates [29].

Several limitations of the study should be noted. First, we utilized a refined cohort within the SEER dataset based on ICD-O codes for clinically relevant histologies – clear cell, papillary and chromophobe. Separate codes do exist that may encompass these histologies. For instance, a search based on the ICD-O code 8312 (“Renal Cell Adeno/Ca”) retrieved a total of 12,155 records – median survival in this cohort was 7 months, leading us to suspect that the search term may encompass a hetergenous array of histologies, such as upper tract urothelial tumors. As such, we felt that it was essential in our analysis to delineate those individuals where clear cell histology had been specified (ICD-O 8310: “Clear Cell Adeno/Ca”), as the preponderance of targeted therapies approved between 2005–2009 (excepting temsirolimus) were assessed in such patients [5]. A second limitation of our study is that the specific nature of systemic therapies rendered is not recorded. Our underlying hypothesis, suggesting that survival is improved in the era of targeted agents, is predicated on the assumption that patients diagnosed from 1992–2004 received immune-based strategies, while patients diagnosed from 2005–2009 received primarily VEGF- and mTOR-directed therapies. Beyond systemic therapy for mRCC, it is possible that improvements in palliative care may contribute to the survival trends observed in mRCC. Enhanced palliative care may ease the burden of toxicities encountered with systemic therapy, and there is an indication that early intervention with palliative care may intrinsically contribute to improved survival in other malignancies [30]. In all likelihood, although targeted therapies first garnered approval in 2005, these agents likely took time to integrate into the standard treatment paradigm for mRCC. Thus, in the earlier part of the second time period, it is still possible that many patients received cytokine therapy. Patients treated around the cutoff employed in this analysis (2005) may have also been exposed to placebo control arms on pivotal phase III studies evaluating targeted agents. It is possible that this may have diluted the difference in survival noted between the cytokine and targeted therapy eras. Finally, as noted previously, the follow-up in the later study period was significantly shorter than in the earlier study period (24.5 *vs* 14.0 months; P<0.0001). Although this discrepancy in duration of follow-up is substantial, this is accounted for by the statistical analysis utilized (i.e., Kaplan-Meier analysis with the log-rank test).

These limitations notwithstanding, our data underscore that progress is being made in the management of mRCC. Population-based studies are critical, and there are few in the available literature. The data assembled by the International mRCC Consortium has provided key insights related to clinical outcome in the era of targeted therapy, and most recently, the group has provided data pertaining to conditional survival [31], [32]. However, extrapolating these data to the population with mRCC at large is challenging because (1) patients in the experience have all received first-line VEGF-directed therapy, and (2) the data is derived largely from experienced academic centers with robust RCC-focused programs. Estimates provided by SEER should provide reassurance that the overarching direction taken in mRCC therapy (most notably, a shift towards targeted therapies) appears to have improved outcomes globally.
